# Combining Network Modeling and Gene Expression Microarray Analysis to Explore the Dynamics of Th1 and Th2 Cell Regulation

**DOI:** 10.1371/journal.pcbi.1001032

**Published:** 2010-12-16

**Authors:** Marco Pedicini, Fredrik Barrenäs, Trevor Clancy, Filippo Castiglione, Eivind Hovig, Kartiek Kanduri, Daniele Santoni, Mikael Benson

**Affiliations:** 1 Istituto per le Applicazioni del Calcolo “Mauro Picone”, Consiglio Nazionale delle Ricerche (CNR), Rome, Italy; 2 The Unit for Clinical Systems Biology, University of Gothenburg, Gothenburg, Sweden; 3 Department of Tumor Biology, Institute of Cancer Research, the Norwegian Radium Hospital, Oslo, Norway; 4 The Institute for Medical Informatics, Rikshospitalet, Oslo University Hospital, Oslo, Norway; 5 Department of Informatics, University of Oslo, Oslo, Norway; 6 Barcelona Institute for Research in Biomedicine (IRB), Barcelona Science Park, Barcelona, Spain; 7 Unit for Pediatric Allergology, Queen Silvia Children's Hospital, Gothenburg, Sweden; New York University, United States of America

## Abstract

Two T helper (Th) cell subsets, namely Th1 and Th2 cells, play an important role in inflammatory diseases. The two subsets are thought to counter-regulate each other, and alterations in their balance result in different diseases. This paradigm has been challenged by recent clinical and experimental data. Because of the large number of genes involved in regulating Th1 and Th2 cells, assessment of this paradigm by modeling or experiments is difficult. Novel algorithms based on formal methods now permit the analysis of large gene regulatory networks. By combining these algorithms with *in silico* knockouts and gene expression microarray data from human T cells, we examined if the results were compatible with a counter-regulatory role of Th1 and Th2 cells. We constructed a directed network model of genes regulating Th1 and Th2 cells through text mining and manual curation. We identified four attractors in the network, three of which included genes that corresponded to Th0, Th1 and Th2 cells. The fourth attractor contained a mixture of Th1 and Th2 genes. We found that neither *in silico* knockouts of the Th1 and Th2 attractor genes nor gene expression microarray data from patients with immunological disorders and healthy subjects supported a counter-regulatory role of Th1 and Th2 cells. By combining network modeling with transcriptomic data analysis and *in silico* knockouts, we have devised a practical way to help unravel complex regulatory network topology and to increase our understanding of how network actions may differ in health and disease.

## Author Summary

Different T helper (Th) cell subsets have an important role in regulating the immune response in inflammatory diseases. Th1 and Th2 cells are thought to counter-regulate each other, and alterations in their balance result in different diseases.This paradigm has been challenged by recent clinical and experimental data. Because of the large number of genes involved in regulating Th1 and Th2 cells, assessment of this paradigm by experiments or modelling is difficult. In this study, we combined novel algorithms for network analysis, *in silico* knockouts, and gene expression microarrays to examine if Th1 and Th2 cells had counter-regulatory roles. We constructed a directed network model of genes that regulated Th1 and Th2 cells through text mining and manual curation. We identified four cycles in the gene expression dynamics, three of which expressed genes that corresponded to Th0 (Th1/Th2 precursor), Th1 and Th2 cells. The fourth cycle contained the expression of a mixture of Th1 and Th2 genes. We found that neither *in silico* knockouts of the Th1 and Th2 attractor genes nor gene expression microarray data from patients and healthy subjects supported a counter-regulatory role of Th1 and Th2 cells.

## Introduction

The immune system is composed of diverse cell populations, for example antigen-presenting cells, T and B lymphocytes as well as effector cells like eosinophils, mast cells and neutrophils. One type of T lymphocytes, called T helper (Th), has an important role in regulating this cellular network. Th cells can be further divided into Th1 and Th2 cells. Th1 and Th2 cells are thought to be mutually inhibitory and also to be involved in different diseases; Th1 cells are associated with autoimmune diseases, while Th2 cells are involved in allergies [1].

Although considered a simplification, the Th1/Th2 dichotomy is supported by a large body of experimental evidence [2]. However, studies of human diseases are more ambiguous in terms of the counter-regulatory roles of Th1 and Th2 cells. We and others have found that allergy, which is mainly thought to be a Th2 disease, can also be associated with Th1 responses [3], [4]. One explanation could be that the Th1/Th2 paradigm is, to a large extent, based on studies of gene interactions in mice which may differ from those in humans, [5]. Another important aspect is that Th1 and Th2 cells interact in complex cellular networks that include several other T-cell subsets and cell types [5]. Ultimately, the balance between Th1 and Th2 cells is complicated to study experimentally, because it is the net result of altered interactions between multiple genes.

Gene expression microarray studies evidence that hundreds of genes are involved in the Th1/Th2 cell differentiation [6]. We and others have found that complex gene expression changes in diseases can be addressed by arranging the genes in networks [7]–[9]. These networks give an overview of the genes that are involved, as well as their interactions, but not the dynamics of network changes that result in phenotypic alterations like, for example, Th1 and Th2 cell differentiation. Recent studies of the dynamics of Th1 and Th2 cell differentiation using *in silico* modeling have to some extent supported a counter-regulatory role of Th1 and Th2 cells [10], [11].

The gene networks used have been based on a relatively small, though relevant, number of genes and interactions. In the present work we applied an algorithm previously developed to analyze large gene regulatory networks to perform *in silico* studies based on a more comprehensive gene network model, which included a larger number of genes [12], [13].

The network was constructed by combining text mining from Medline (www.pubmed.com) based on seed genes and protein interaction data, with manual annotation. The aim of our study was to examine if the so-constructed network model was compatible with a counter-regulatory role of Th1 and Th2 cells from healthy humans as well as patients with different inflammatory diseases.

To achieve this we studied the effects of *in silico* knockouts on the model dynamics [14], together with analyses of gene expression microarray studies of T-cells from healthy controls and patients with different inflammatory diseases.

## Results

### Definition of a network model of Th1 and Th2 differentiation

**Figure 1:**
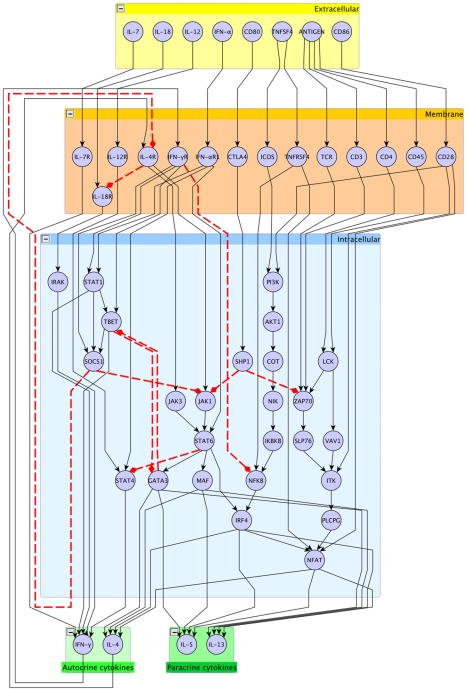
Systemic view of the gene regulatory network model including relevant genes or transcription factors for Th1 Th2 cell differentiation. Black edges depict positive regulation; red edges negative regulations.

We defined a gene regulatory network (GRN) model of the genes involved in Th1 and Th2 cell differentiation based on manual annotation and automated data mining of Medline abstracts. To ease inspection, this gene regulatory network was organized into four layers according to the sub-cellular localization of the genes (see Figure 1). Another reason for this exercise was to enable the network for usage in agent-based models, as in [15].

The extracellular layer included cytokines (IL-7, TNFSF4, IFN-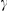, IL-12 and IL-18), the antigens, as well as two membrane-receptors expressed on antigen-presenting cells, namely CD80 and CD86. The membrane layer consisted of the T-cell receptor and cytokine receptors. The intracellular layer included signaling molecules as well as transcription factors. Finally, an extra-cellular layer consisted of autocrine cytokines (IL-4 and IFN-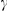) and paracrine cytokines (IL-5 and IL-13).

### Characterization of the attractors of the network

Gene regulatory networks (GRNs) can be represented as graphs where nodes represent genes that are either active or inactive. The state of the network is given by the combination of the activation state of all genes. Starting from a certain state, the upcoming configuration is computed by applying synchronously an updating rule. In general, since the number of possible states is *finite* (*i.e.*, 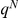 if 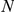 is the number of nodes, and 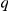 is the number of possible values of a node), and the dynamics is deterministic, then from a given initial state, the network can only evolve towards a limit cycle (*i.e.*, *attractor*) of length one or more (up to 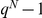).

In what follows, we go after Kauffman [15] by identifying the attractors of the network dynamics as differentiation phases of the cell, and the transformations between attractors as pathways of cell differentiation.

Using the algorithm in [12] (briefly discussed in the Materials and Methods section), we found that the GRN dynamics was characterized by four attractors, three of which corresponded to known Th subsets, namely Th0, Th1 and Th2. The remaining attractor, which we named ThX, contained both Th1 and Th2 genes (see Table 1).

**Table 1:** The attractors of the boolean network modeling Th1/Th2 differentiation.

Attractor	Active genes
Th0	None
Th1	IFN- 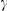 , IFN- 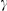 R, SOCS1, STAT1, TBET
Th2	GATA3, IL-13, IL-4, IL-4R, IL-5, IRF4, JAK1, JAK3, MAF,
	NFAT and STAT6
ThX	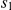 : IFN- 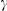 , IL13, IL-4, IL-5, JAK3, NFAT and SOCS1
	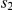 : IFN- 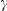 R, IL13, IL-4, IL-5 and STAT6
	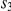 : GATA3, IL-4R, IRF4, MAF, SOCS1, STAT1 and TBET

ThX is the non-Th1-nor-Th2 attractor, consisting of a cycle composed by the three states 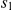, 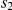 and 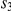.

The Th1 and Th2 attractors contained either Th1 or Th2 genes, an observation that was compatible with a counter-regulatory role of Th1 and Th2 cells. For example, the Th1 attractor contained the transcription factor TBET, which has been experimentally shown to induce the Th1 cytokine IFN-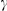 and inhibit the Th2 transcription factor GATA3, which, in turn, induces the Th2 cytokine IL-4. Conversely, GATA3 inhibits TBET and IFN-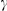. Thus, the two transcription factors TBET and GATA3 play a key role in the counter-regulatory interaction between Th1 and Th2 [5]. However, the mixture of Th1 and Th2 genes in the ThX attractor did not agree with a counter-regulatory role between Th1 and Th2 cells. In particular, the state 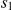 contained both IFN-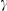 and IL-4, while the state 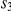 contained both TBET and GATA3 (Table 1). This suggested that the dynamics of the network had an important role in regulating the balance between Th1 and Th2 cells. This may correspond, *in vivo*, to the situation in which antigenic stimulation may be temporary or persisting, and result in different inflammatory responses [16].

### *In silico* knockouts to model the dynamics of the network

We performed single gene *in silico* knockout experiments for all genes in the network, in order to monitor the behaviour of the attractors. In so doing, we distinguished two different settings, corresponding to a different activation modality of the input nodes (*i.e.*, those contained in the yellow box of Figure 1): *temporary*-stimulation and *persisting*-stimulation. In temporary stimulation we examined the effects of an impulse-like stimulation of the input genes, which means that those genes were considered active for a short and transient period of time, and were set off thereafter. In persisting stimulation instead, inputs were set on or off throughout the observation period. Persisting stimulation is equivalent to introducing self-loops on the input nodes of the GRN.

We computed the number of attractors for each single-gene knockout and for both activation modalities. We found that the median number of attractors per knocked out gene was 4 (range 3–9) for temporary stimulation whereas it was 604 (range 322–1664) for persisting stimulation, (Table 2).

**Table 2:** Number of attractors in knock-out networks.

	Temporary Stimulation			Sustained Stimulation		
knock-out gene	attractors	(static/dynamical)	max	attractors	(static/dynamical)	max
COT	4	(3/1)	3	1186	(898/288)	3
GATA3	3	(3/0)	1	322	(322/0)	1
IKBKB	4	(3/1)	3	594	(450/144)	3
IRAK	4	(3/1)	3	612	(452/160)	3
IRF4	9	(3/6)	5	604	(450/154)	5
ITK	4	(3/1)	3	1188	(900/288)	3
JAK1	4	(3/1)	3	594	(450/144)	3
JAK3	3	(3/0)	1	560	(432/128)	2
LCK	4	(3/1)	3	1187	(899/288)	3
MAF	4	(3/1)	3	594	(450/144)	3
NFAT	9	(3/6)	5	604	(452/152)	5
NFKB	4	(3/1)	3	594	(450/144)	3
NIK	4	(3/1)	3	1186	(898/288)	3
PI3K	4	(3/1)	3	1186	(898/288)	3
PLCPG	4	(3/1)	3	596	(452/144)	3
SHP1	4	(3/1)	3	594	(450/144)	3
SLP76	4	(3/1)	3	594	(450/144)	3
SOCS1	8	(5/3)	3	978	(594/384)	3
STAT1	7	(3/4)	6	1154	(482/672)	7
STAT4	4	(3/1)	3	612	(452/160)	3
STAT6	6	(3/3)	3	1664	(1088/576)	3
TBET	7	(3/4)	6	358	(322/36)	6
VAV1	4	(3/1)	3	595	(451/144)	3
ZAP70	4	(3/1)	3	1186	(898/288)	3

Number of attractors for the sustained and temporary stimulation; we give also the number of attractors which are of length one (*static equilibrium*) or of length greater than 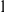 (*dynamical equilibrium*); moreover, we indicate the maximal length of attractors.

Therefore, as a first observation we noted that, similarly to *in vivo* stimulation, the network dynamics differed greatly between temporary and persisting stimulation. Next, we proceeded to examine the counter regulatory dynamics of the Th1 and Th2 cells. This was done by testing the effects of *in silico* knockouts of intra-cellular genes in the Th0, Th1, Th2 and ThX attractors. We started by knocking out TBET and GATA3. If TBET and GATA3 were counter-regulatory, knocking out TBET would be expected to result in attractors mainly containing IL-4, but not IFN-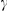, while the opposite would be expected after knocking out GATA3.

**Figure 2:**
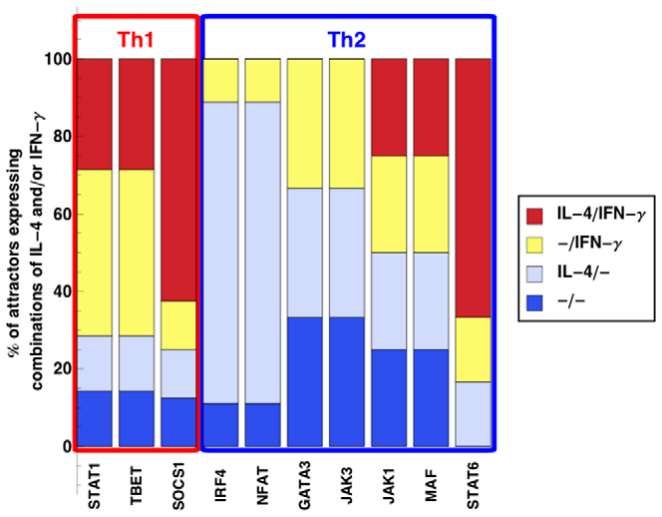
Number of attractors as the result of *in silico* knockout experiments, in the temporary stimulation activation modality. Stacked bars represent the percentage of attractors expressing combinations of IL-4 and/or IFN-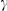.

Firstly, we applied the temporary stimulation activation modality (Figure 2). Knocking out TBET resulted in attractors that contained both IL-4 and IFN-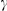, either IFN-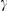 or IL-4, as well as attractors without IL-4 and IFN-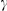. Knocking out IL-4 resulted in attractors that contained either IFN-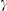 or IL-4, as well as attractors without IL-4 and IFN-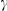.

**Figure 3:**
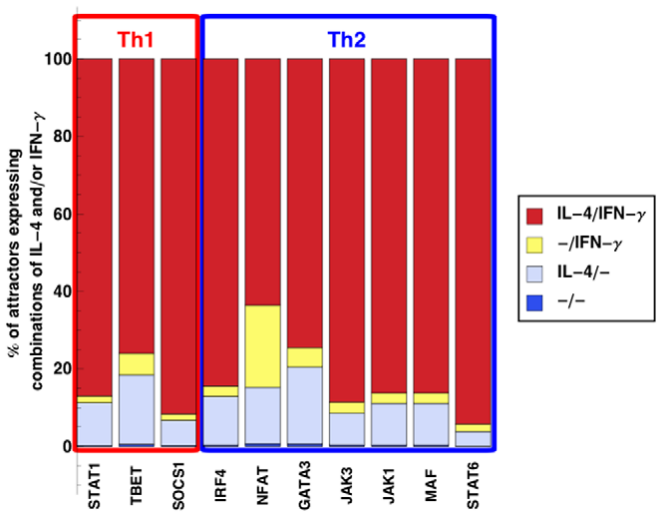
Number of attractors as the result of *in silico* knockout experiments, in the persisting stimulation activation modality. Stacked bars represent the percentage of attractors expressing combinations of IL-4 and/or IFN-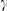.

On the other hand, knocking out the same genes but applying the persisting stimulation activation modality mainly resulted in attractors containing both IL-4 and IFN-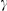 (Figure 3).

For both temporary and persisting stimulation, the knockout of other transcription factors that regulated Th1 and Th2 cells, namely IRF4, MAF, NFAT, STAT1 and STAT6, also resulted in attractors that contained IL-4 and IFN-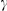, either alone or in combination. Thus, the balance between Th1 and Th2 cells was regulated by several transcription factors, and not only by TBET and GATA3.

To summarize, these findings were not compatible with a strictly counter-regulatory role of neither TBET nor GATA3 or any of the other transcription factors.

### Analysis of relations between *in silico* and *in vitro* findings in human T-cells in health and disease

We proceeded to examine how the *in silico* findings related to *in vitro* studies of T-cells from healthy controls and patients with different T-cell related diseases. We downloaded several sets of gene expression microarray data from the public domain to test whether Th1 and Th2 genes were inversely correlated in T-cell related diseases.

If Th1 and Th2 cells are antagonists we would expect inverse relations between genes in the Th1 and Th2 attractors. If so, the expression levels of those genes would be negatively correlated. Instead of this, we found a highly significant positive correlation between the ratios of differentially expressed Th1-associated genes and Th2-associated genes (Pearson correlation coefficient 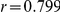, 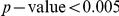).

Thereafter, we analyzed the correlations between all gene pairs in the model that, based on the literature, were considered to inhibit each other. This analysis showed that all gene pairs were positively correlated but one (see Table 3).

**Table 3:** Results of Pearson's correlation test of inhibitory gene pairs.

Gene Pair	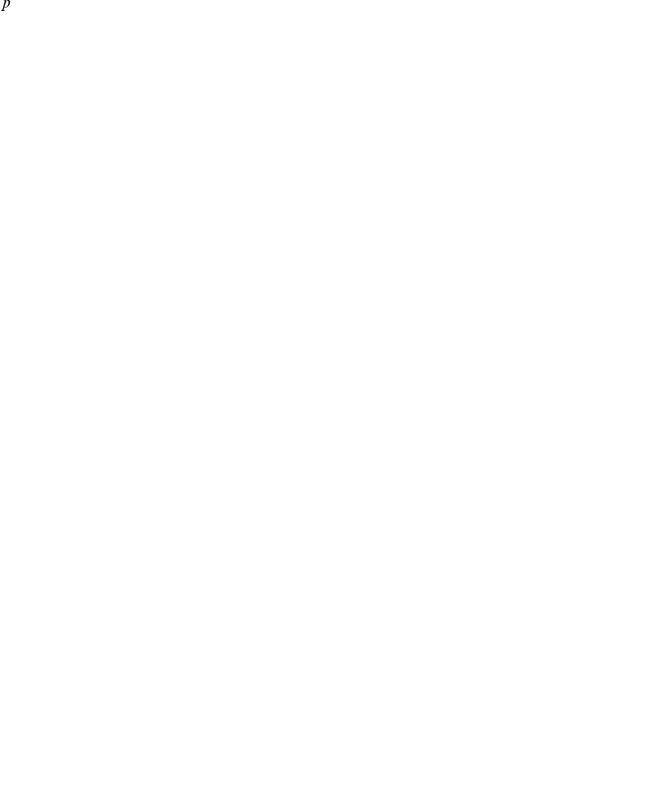 -value	correlation 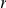
GATA3 - TBET	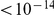	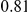
SHP1 - JAK1	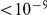	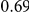
IL-4R - IL18R	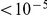	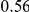
SOCS1 - IL-4R	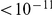	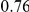
SOCS1 - JAK3	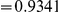	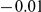
STAT6 - STAT4	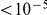	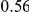
IFN- 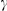 - IL-4	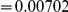	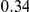

This included the signature Th1 and Th2 genes TBET and GATA3, which showed the most significant positive correlation (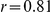, 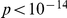) as well as IFN-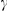 and IL-4 (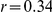, 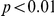).

## Discussion

Because of the large number of proteins involved in Th cell differentiation, alterations in the balance between those proteins are not easily studied experimentally. Computational modeling provides an attractive alternative to study the dynamics of Th1 and Th2 cell regulation and has previously been employed for this purpose by us and others [10], [11], [17].

Such models have supported a counter-regulatory role of Th1 and Th2 cells, but were based on a relatively limited number of genes and did not include comparisons with biological data. In this report, we aimed to examine if Th1 and Th2 cells were counter-regulatory by combining modeling, *in silico* knockouts and gene expression microarray analyses of human T cells in health and disease. We constructed a network model of the proteins involved in Th cell differentiation by manual curation of proteins associated with Th1 and Th2 cells, and that had been identified as relevant through automated text mining of the medical literature. This resulted in a significantly more comprehensive model compared to previous versions.

Analysis of the dynamics of that model showed that it contained four attractors, two of which corresponded to the Th1 and Th2 subsets. These contained the Th1 and Th2 specific transcription factors TBET and GATA3, respectively. This was compatible with a counter-regulatory role of these attractors. However, the fourth attractor, which we named ThX, contained a mixture of Th1 and Th2 proteins, including TBET and GATA3. This did not agree with a counter-regulatory role of these transcription factors.

Furthermore, we extended our analysis by *in silico* knockout experiments of TBET and GATA3. We reasoned that if the two were counter-regulatory, then knocking out TBET would result in attractors mainly containing IL-4, while knocking out GATA3 would result in attractors mainly containing IFN-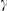. Whereas this was true for GATA3, it was not the case for TBET.

In fact, knockout of either TBET or the other Th1 and Th2 attractor proteins mainly resulted in attractors containing both IFN-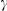 and IL-4. Afterthat, we examined the expression of Th1 and Th2 attractor genes in microarray studies of eleven T cell diseases, namely autoimmune, infectious and oncological diseases.

In most of these, the expression of Th1 and Th2 attractor genes increased concurrently, rather than in an opposing pattern. Moreover, we found that genes in the network model that were thought to inhibit each other based on experimental studies, were in fact positively correlated. This was particularly true for TBET and GATA3 which are thought to have a key role for the counter-regulation of Th1 and Th2 cells. It is of note that the interactions in the model were chosen based on experimentally validated functions and interactions in Th cells. In many cases those experiments were performed using polarizing cytokines and T cell receptor stimulants. This is likely to result in more homogenous Th cell responses than those seen *in vivo*. In the latter case Th cells are activated by antigen-presenting cells which process the antigens to peptides, subtle variants of which may have different effects on Th cells. In addition, different doses and timing of antigen exposure play an important role in the Th cell activation and differentiation process. The effects of timing was reflected by the results in our study; temporary and persistent stimulation had profound effects on the network dynamics of these processes.

Moreover, the activation involves a complex and variable mixture of proteins. Taken together, it is possible that this complexity may result in a mixture of Th1 and Th2 cells responses, rather than one of the two. The ThX attractor may correspond to such a mixed or transitional response. This is consistent with the increasing recognition that Th cell phenotypes are plastic rather than discrete [2]. This recognition resulted from experimental and clinical studies that show overlap between genes considered to be Th1 and Th2 genes [18], [19].

Our analyses of gene expression microarray data from human T cells in health and disease lend further support to Th plasticity. From an *in vivo* perspective, this plasticity allows fine-tuned responses to a constant exposure of different antigens at different time points and doses.

It is also of note that *in vivo* Th1 and Th2 differentiation may be affected by many other T cell subsets, of which an increasing number have been recognized. Moreover, epithelial cells, mast cells and eosinophils release cytokines that affect the differentiation process. Ideally, simultaneous analysis of networks representing those cells and subsets would yield an understanding not only of Th1 and Th2 cells, but comprehensive models of the cellular networks that underlie immunological diseases. Improved methodologies, such as single cell RNA sequencing may make such models feasible in the near future.

A limitation is that our model is that the underlying biological data is mainly qualitative. Thus, the model is based on synchronous updating and does not take into account quantitative or time-dependent changes. Others have shown that asynchronous updating may have different effects on attractors [20]–[22]. An interesting future research direction is to perform time series experiments of Th1 and Th2 cells using gene expression microarrays. Using such data it may be possible to improve our model both with regards to quantitative and time-dependent changes and also make predictions which can be validated with other biological methods, such as measuring Th1 and Th2 cytokines on the protein level.

In summary, our findings, both based on *in silico* modeling and analysis of T cells from human diseases agree with Th1 and Th2 cells having complex and possibly synergistic, rather than counter-regulatory roles.

## Materials and Methods

### Identification of genes for the network model of Th1/Th2 cell differentiation

We employed a step-wise procedure to define the set of relevant genes for the differentiation of Th cells into the Th1 and Th2 phenotypes.

Firstly, we identified two different sets of genes as a primary source: i) 17 genes from a previous network model [10]; ii) a set of 17 genes determined in a gene expression microarray study of polarized Th1 and Th2 cells by [6]. All these 34 genes were used as seed genes. Then we retrieved the first order neighbors of these seed genes and their connections in the BioGrid database (www.biogrid.org). Successively, the connection among the proteins of the first order neighbors were retrieved. Among all the genes retrieved thus far, we selected only those associated to the Gene Ontology term (www.geneontology.org) “T cell differentiation”.

More specifically, the genes co-cited in the millions Medline abstracts together with this term were retrieved. This resulted in a set of 403 genes, that was further slimmed down and used to construct a manually annotated directed graph of gene interactions relevant for Th1 and Th2 cell differentiation. This was made by using the T-cell receptor pathway in the KEGG database as a template (www.genome.jp/kegg/pathway.html). Genes that were part of that pathway and had well-characterized and experimentally verified functions relevant for Th1 and Th2 cell differentiation were selected for the final network model. A detailed description of each interaction in the network, together with 126 supporting references is given in Text S1. It is also of note, that the network model was independent of the gene expression microarray experiments, which are described below (none of the published abstracts pertaining to those experiments contained co-cited genes that were included in the model).

### Boolean networks as a model of Genetic Regulatory Networks

Given a GRN, the number of attractors of the network dynamics is, in general, not effectively computable since the number of states of the network grows exponentially with 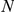. It is not even possible to effectively calculate the initial states of the network that will eventually fall in the basin of attraction of a specified limit cycle. When the number of genes is large, the explicit computation of the dynamics becomes impractical as the number of states the network can assume increases exponentially with the number of nodes. In the worst case the algorithm needs to store the complete description of the state transition graph and therefore the exhaustive study is feasible only when the number of nodes is small [10], [23]. Just to give an idea, for a network with 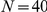 nodes, one needs about 6 Terabytes to store the state-transition graph of the network. In our case, with 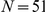, it would require about 7 Petabytes of storage.

In recent studies, formal methods such as *bounded model-checking technique* or *reduced order binary decision diagrams* have been employed in the study of attractors of Boolean and multivalued networks, see Dubrova *et al.*, Garg *et al.*, and Chaves *et al.* [12], [21], [24]–[26]. These formal methods have a potential to handle large networks. In particular we used Dubrova's algorithm based on a solver for the *satisfiability* problem (SAT) which from the logical structure of the network infers the possible attractors. In simple words, the network can be seen a Boolean circuit and its attractors can be computed by using methods and largely optimised algorithms coming from modeling of *Very Large Scale Integration* (VLSI) circuits.

What is special about formal methods approach is that it enables to find attractors of large networks. The idea behind the search algorithm is that, by using *symbolic* computation, it is possible to unfold the dependencies between nodes that are linked together and to compose the update function as a relation among the states (activation/inhibition) of the genes/nodes. Then the algorithm uses the SAT solver to determine the values of the states that *evaluate to true* the relation. This process is then repeated until all attractors are identified.

We specified the network as the set of rules 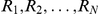, each one representing the activatory or inhibitory relation between genes. For example, if rule 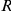 stems for the activation of gene 
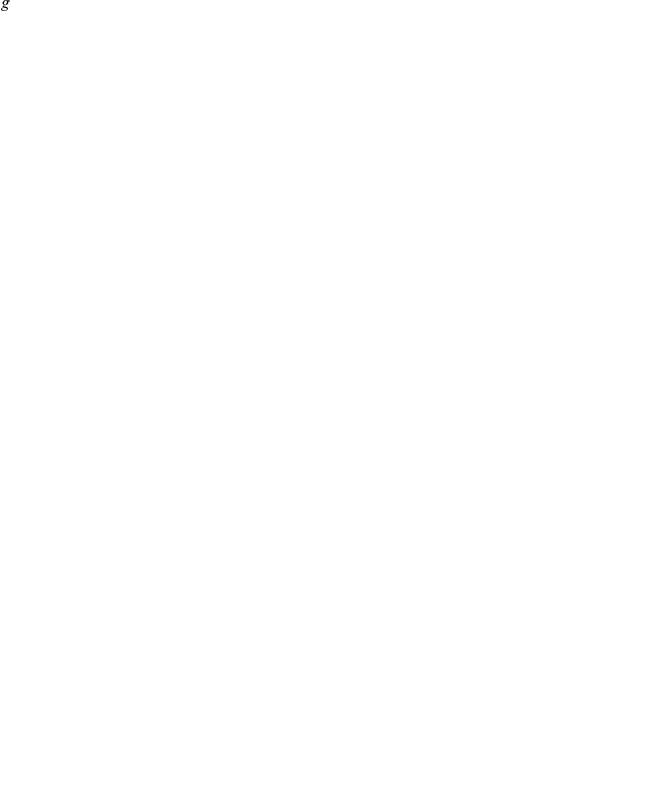
, and is determined by the activators 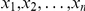 and inhibitors 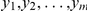 (activators and inhibitors are generically called regulators), then it can be written as 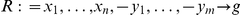, where conventionally the subset of inhibitors are tagged with a minus sign.

Analogously to [10], [12], the time is discrete and the activation states of the genes are changed simultaneously (*i.e.*, synchronous update). At each time step 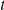, the value of the gene 
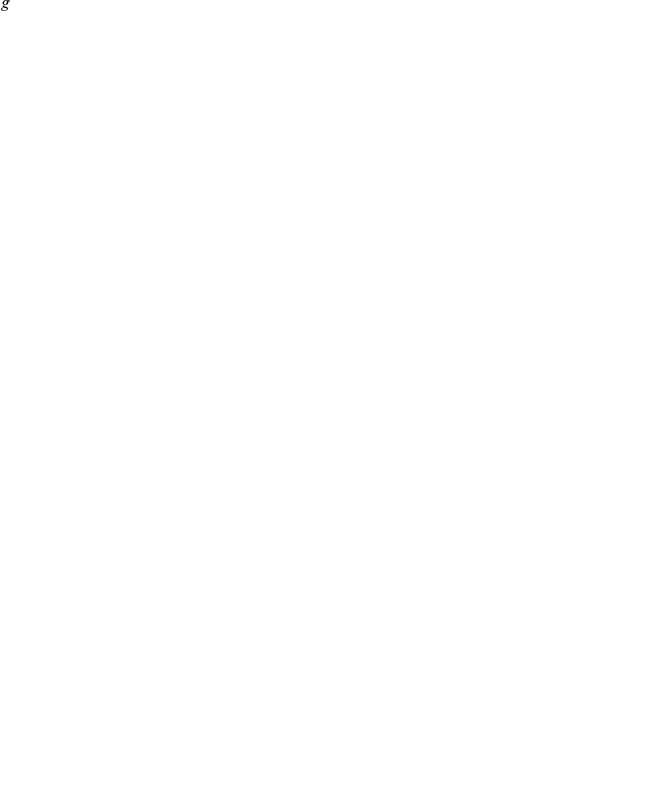
 is denoted by the same gene name 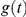. The successive value of gene 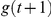 is
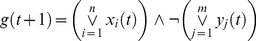
(1)where 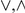 and 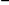 denote the logical operators *and*, *or* and *not* respectively. The rule in Equation 1 states that for a gene to be activated, at least one activator and no inhibitors must be active [10 12, 27].

In our specific case we had a set of 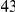 rules involving 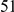 genes, that was the result of data mining and manual annotation. These are listed in Table 4. The network so specified was compiled in other formats, in particular GML (Graph Modeling Language), which is used in several applications specialized in graphical layout, and CNET which is the input form accepted by the algorithm to compute the attractors. Whereas the GML output was based simply on activation/inhibition network links, in the CNET format we had to specify the updating function for each node.

**Table 4:** Specification rules for activation/inihibition links of the network in Figure 1.

IRF4, NFAT, MAF, GATA3	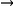	IL-13
IRF4, NFAT, MAF, GATA3	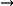	IL-5
IFN- 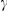 R, -GATA3, STAT1	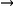	TBET
IL-7R, TBET, STAT4,STAT1,IRAK	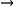	IFN- 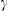
STAT6	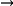	MAF
STAT6, -TBET	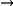	GATA3
IL-7	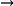	IL-7R
IL-18, -IL-4R	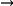	IL-18R
IL18R	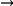	IRAK
IFN- 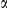 R1, IFN- 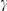 R	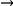	STAT1
IFN- 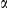	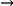	IFN- 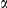 R1
IFN- 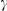	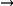	IFN- 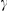 R
IL-4, -SOCS1	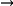	IL-4R
IRF4, NFAT, MAF, GATA3	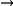	IL-4
CD80	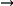	CTLA4
CTLA4	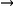	SHP1
CD45, CD4	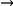	LCK
TCR, CD3, -SHP1,LCK	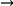	ZAP70
ZAP70	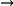	SLP76
LCK	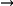	VAV1
CD28, VAV1, SLP76	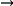	ITK
ITK	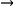	PLCPG
ANTIGEN	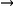	CD4
ANTIGEN	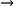	TCR
ANTIGEN	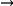	CD3
ANTIGEN	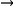	CD45
TNFSF4	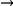	TNFRSF4
-IFN- 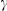 R, TNFRSF4, IKBKB	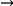	NFKB
STAT6, NFKB	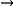	IRF4
CD28, TNFRSF4, PLCPG, IRF4	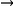	NFAT
CD28, ICOS	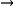	PI3K
PI3K	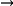	AKT1
AKT1	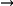	COT
COT	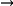	NIK
NIK	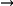	IKBKB
CD86	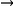	CD28
IL-4R, -SHP1,-SOCS1	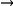	JAK1
IL-4R	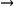	JAK3
IFN- 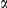 R1, JAK1,JAK3	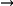	STAT6
IL12R, IFN- 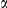 R1, -STAT6	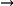	STAT4
IFN- 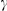 R, STAT1, TBET	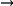	SOCS1
IL-12	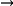	IL-12R
TNFSF4	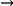	ICOS

The last part of this work was the systematic characterization of the networks obtained by knocking out genes one at a time. As a consequence of these *in silico* knockout experiments we anticipated two results: a) to identify the set of genes which are pivotal to the Th1/Th2 differentiation; b) to spot subsets of co-expressed genes belonging to the attractors, since from analysis of microarray data we expected these genes to be correlated.

Changes in the set of the attractors were used to highlight relevant nodes. As a first approximation, differences in the mere number of attractors were considered; if a node did not affect the number of attractors, then from the point of view of the dynamics it was considered irrelevant.

### Compilation and analysis of gene expression microarray data

To examine whether Th1 and Th2 gene activation patterns denoted two opposed pathways, gene expression data were downloaded from Gene Expression Omnibus (http://www.ncbi.nlm.nih.gov/geo/). Datasets were selected based on the criteria that they i) measured mRNA expression from CD4+ cells from healthy controls or patients with T-cell related diseases (*e.g.*, SLE) and ii) that there were at least 5 samples per disease or per controls, (Table 5).

**Table 5:** Gene expression microarray datasets downloaded from the Gene Expression Omnibus repository.

GEO Accession Number	Disorder
GSE4588	Systemic Lupus Erythematosus (SLE), Rheumatoid Arthritis (RA)
GSE6740	HIV
GSE8835	B cell chronic lymphocytic leukemia (CLL)
GSE9927	Type I HIV (HIV-I)
GSE10586	Type 1 Diabetes (T1D)
GSE12079	Hypereosinophilic syndrome
GSE13732	Clinically Isolated Syndrome - Multiple Sclerosis
GSE14317	Adult T-cell leukemia/lymphoma (ATL)
GSE14924	Acute Myeloid Leukaemia (AML)
GSE17354	Adenosine deaminase (ADA) - Severe combined immunodeficiency (SCID) (Therapy treated)

Differentially expressed genes between patients and controls in each disease were determined using the unpaired Student's t-test. Genes with a significance 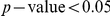 were considered differentially expressed.

In order to examine if the differentially expressed genes in the Th1 and Th2 attractors were negatively or positively correlated we performed the following analyses: for each disease, the ratio between differentially expressed genes in the Th1 attractor and all genes in the Th1 attractor was computed. This analysis was repeated for the Th2 attractor genes. It resulted in a list of ratios for each attractor and for each disease. The Pearson correlation coefficient between those ratios was then computed.

To test if gene pairs in the network model that had counter-regulatory relationships were also negatively correlated, microarray data belonging to healthy controls in each dataset was pooled and Pearson correlation coefficients were calculated for all the gene pairs with counter-regulatory relationships.

## Supporting Information

Text S1 References for interactions. In this document we present references supporting interactions introduced in our model network.
(0.12 MB PDF)
