# Supplementary material for: Combining Network Modeling and Gene Expression Microarray Analysis to Explore the Dynamics of Th1 and Th2 Cell Regulation
Source: PLoS Comput Biol. 2010 Dec 16;6(12):e1001032. doi: 10.1371/journal.pcbi.1001032 (PMC3002992; doi:10.1371/journal.pcbi.1001032)
Supplement: Text S1 — References for interactions. In this document we present references supporting interactions introduced in our model network. (0.12 MB PDF) [file pcbi.1001032.s001.pdf]

A description of the manual curation of the nodes and interactions underlying the network model

From a general perspective the curation process was guided by recent reviews and original articles relating to Th1/Th2 cell differentiation *in vitro* and *in vivo* [1-3] and its transcriptional regulation [4, 5] as well as current discussions about Th plasticity and Th cell interactions [6-9]. We also considered modelling and network studies of T cell differentiation by us and others [10-13].

A specific description of each node and interaction in the network model is given below, followed by a separate list of 126 references:

A specific description of each node and interaction in the network

The induction of IL4 expression by GATA3, MAF, NFAT and IRF4, *in vitro* as well as *in vivo* has been described in several reports: GATA3 [4, 14, 15], NFAT [16-18], MAF [19-21] and IRF4 [17, 22]. IL5 and IL13 are also induced by GATA3, MAF, NFAT and IRF4: IL5 and GATA3 [23-25], MAF [26], NFAT [27, 28], IRF4[17]. IL13 GATA3[29] MAF [30] NFAT [27, 31] IRF4[22]. IL4 induces the expression of IL4R [32, 33].

IFNG is induced by IL7R[34], TBET[35], STAT4 [36, 37],STAT1[38], and IRAK[39]. IFNG and IFNGR increases transcription of TBET [40, 41], as does STAT1 [42-44], while GATA3 inhibits TBET[45]. IFNAR1 activates STAT1 [46-48], as does IFNGR IFN-γR [44, 49, 50]. IFNA activates IFNAR1[51]. IFNG induces expression of IFNGR [52, 53].

STAT6 activates MAF [54]and GATA3 [54-56], while TBET inhibits GATA3 [45, 57-59]. IL18 activates IL18R[60], and IL18R activates IRAK[61].

IL7 activates IL7R[62]. IL4 increases expression of IL4R [33], while SOCS1 has the opposite effect[63]. CD80 increases recruitment of CTLA4 to the T cell synapse[64]. CTLA4 activates SHP1[65]. CD45 [66-68], and CD4 [69, 70] activates LCK. LCK activates VAV1 [71-73]. TCR, [74-76], CD3 [74, 77, 78] and LCK activates ZAP70 [74, 79], while SHP1[80] inhibits ZAP70. ZAP70 increases binding of SLP76 to other proteins in the TCR pathway[74, 81]. CD28[82, 83], VAV1 and SLP76 [75, 84]activates ITK. ITK activates PLCPG[75, 85, 86].

TNFSF4 activates TNFRSF4[87]. TNFRSF4 [88]and IKBKB [89, 90]activate NFKB. STAT6 [91]and NFKB [92]activates IRF4. CD28[19, 93], TNFRSF4[94], PLCPG, [95]and IRF4 [17] either are required for or increases activation of NFAT.

CD28 [82, 96, 97] and ICOS[98, 99] increases activation of PI3K. PI3K increases activation of AKT1[100, 101]. AKT1 increases activation of COT[102]. NIK increases activation of IKBKB[103-105]. CD86 increases activation of CD28[106].

IL-4R increases activation of JAK1[32, 107], while SHP1 [108] and SOCS1 of [109-111] decreases activation of JAK1. IL-4R activates JAK3 [32, 112, 113]. IFN-αR1[47, 114], JAK1 [115] and JAK3 [107, 115, 116] activates STAT6. IL12R [44, 117, 118], IFN-αR1 [114, 119] activates STAT4, while STAT6 [120, 121] inhibits STAT4. IFNGR[122], STAT1[123-125] and TBET [126] increase expression of SOCS1. IL12 increases expression of IL12R [58].

References

1. Paul, W.E. and J. Zhu, *How are T(H)2-type immune responses initiated and amplified?* Nat Rev Immunol, 2010. **10**(4): p. 225-35.
2. O'Shea, J.J. and W.E. Paul, *Mechanisms underlying lineage commitment and plasticity of helper CD4+ T cells.* Science, 2010. **327**(5969): p. 1098-102.
3. Reiner, S.L., *Decision making during the conception and career of CD4+ T cells.* Nat Rev Immunol, 2009. **9**(2): p. 81-2.
4. Ho, I.C., T.S. Tai, and S.Y. Pai, *GATA3 and the T-cell lineage: essential functions before and after T-helper-2-cell differentiation.* Nat Rev Immunol, 2009. **9**(2): p. 125-35.
5. Rautajoki, K.J., et al., *An insight into molecular mechanisms of human T helper cell differentiation.* Ann Med, 2008. **40**(5): p. 322-35.
6. Zhu, J. and W.E. Paul, *CD4+ T cell plasticity-Th2 cells join the crowd.* Immunity, 2010. **32**(1): p. 11-3.
7. Garber, K., *Immunology's quiet upheaval.* Nat Biotechnol, 2009. **27**(8): p. 687-9.
8. Rowell, E. and C.B. Wilson, *Programming perpetual T helper cell plasticity.* Immunity, 2009. **30**(1): p. 7-9.
9. Gadina, M. and J.J. O'Shea, *Immune modulation: Turncoat regulatory T cells.* Nat Med, 2009. **15**(12): p. 1365.
10. Mendoza, L., *A network model for the control of the differentiation process in Th cells.* Biosystems, 2006. **84**(2): p. 101-14.
11. Garg, A., et al., *Synchronous versus asynchronous modeling of gene regulatory networks.* Bioinformatics, 2008. **24**(17): p. 1917-25.
12. Georgescu, C., et al., *A gene regulatory network armature for T lymphocyte specification.* Proc Natl Acad Sci U S A, 2008. **105**(51): p. 20100-5.
13. Santoni, D., M. Pedicini, and F. Castiglione, *Implementation of a regulatory gene network to simulate the TH1/2 differentiation in an agent-based model of hypersensitivity reactions.* Bioinformatics, 2008. **24**(11): p. 1374-80.
14. Zhu, J., et al., *GATA-3 promotes Th2 responses through three different mechanisms: induction of Th2 cytokine production, selective growth of Th2 cells and inhibition of Th1 cell-specific factors.* Cell Res, 2006. **16**(1): p. 3-10.
15. McInnes, I.B. and G. Schett, *Cytokines in the pathogenesis of rheumatoid arthritis.* Nat Rev Immunol, 2007. **7**(6): p. 429-42.
16. Chuvpilo, S., et al., *Multiple closely-linked NFAT/octamer and HMG I(Y) binding sites are part of the interleukin-4 promoter.* Nucleic Acids Res, 1993. **21**(24): p. 5694-704.
17. Rengarajan, J., et al., *Interferon regulatory factor 4 (IRF4) interacts with NFATc2 to modulate interleukin 4 gene expression.* J Exp Med, 2002. **195**(8): p. 1003-12.
18. Sisk, T.J., et al., *MHC class II transactivator inhibits IL-4 gene transcription by competing with NF-AT to bind the coactivator CREB binding protein (CBP)/p300.* J Immunol, 2000. **165**(5): p. 2511-7.
19. Nurieva, R.I., *Regulation of immune and autoimmune responses by ICOS-B7h interaction.* Clin Immunol, 2005. **115**(1): p. 19-25.
20. Lieberson, R., et al., *Tumor necrosis factor receptor-associated factor (TRAF)2 represses the T helper cell type 2 response through interaction with NFAT-interacting protein (NIP45).* J Exp Med, 2001. **194**(1): p. 89-98.

21. Ho, I.C., et al., *The proto-oncogene c-maf is responsible for tissue-specific expression of interleukin-4.* Cell, 1996. **85**(7): p. 973-83.
22. Hu, C.M., et al., *Modulation of T cell cytokine production by interferon regulatory factor-4.* J Biol Chem, 2002. **277**(51): p. 49238-46.
23. Ranganath, S., et al., *GATA-3-dependent enhancer activity in IL-4 gene regulation.* J Immunol, 1998. **161**(8): p. 3822-6.
24. Patel, D.R., M.H. Kaplan, and C.H. Chang, *Altered Th1 cell differentiation programming by CIITA deficiency.* J Immunol, 2004. **173**(9): p. 5501-8.
25. Zheng, W. and R.A. Flavell, *The transcription factor GATA-3 is necessary and sufficient for Th2 cytokine gene expression in CD4 T cells.* Cell, 1997. **89**(4): p. 587-96.
26. Ho, I.C., D. Lo, and L.H. Glimcher, *c-maf promotes T helper cell type 2 (Th2) and attenuates Th1 differentiation by both interleukin 4-dependent and -independent mechanisms.* J Exp Med, 1998. **188**(10): p. 1859-66.
27. Rao, A., C. Luo, and P.G. Hogan, *Transcription factors of the NFAT family: regulation and function.* Annu Rev Immunol, 1997. **15**: p. 707-47.
28. Liu, J., K. Arai, and N. Arai, *Inhibition of NFATx activation by an oligopeptide: disrupting the interaction of NFATx with calcineurin.* J Immunol, 2001. **167**(5): p. 2677-87.
29. O'Garra, A. and N. Arai, *The molecular basis of T helper 1 and T helper 2 cell differentiation.* Trends Cell Biol, 2000. **10**(12): p. 542-50.
30. Tournier, J.N., et al., *Anthrax toxins: a weapon to systematically dismantle the host immune defenses.* Mol Aspects Med, 2009. **30**(6): p. 456-66.
31. Macian, F., C. Garcia-Rodriguez, and A. Rao, *Gene expression elicited by NFAT in the presence or absence of cooperative recruitment of Fos and Jun.* EMBO J, 2000. **19**(17): p. 4783-95.
32. Nelms, K., et al., *The IL-4 receptor: signaling mechanisms and biologic functions.* Annu Rev Immunol, 1999. **17**: p. 701-38.
33. So, E.Y., H.H. Park, and C.E. Lee, *IFN-gamma and IFN-alpha posttranscriptionally down-regulate the IL-4-induced IL-4 receptor gene expression.* J Immunol, 2000. **165**(10): p. 5472-9.
34. Mobini, R., et al., *A module-based analytical strategy to identify novel disease-associated genes shows an inhibitory role for interleukin 7 Receptor in allergic inflammation.* BMC Syst Biol, 2009. **3**: p. 19.
35. Tong, Y., T. Aune, and M. Boothby, *T-bet antagonizes mSin3a recruitment and transactivates a fully methylated IFN-gamma promoter via a conserved T-box half-site.* Proc Natl Acad Sci U S A, 2005. **102**(6): p. 2034-9.
36. Nakanishi, K., et al., *Interleukin-18 is a unique cytokine that stimulates both Th1 and Th2 responses depending on its cytokine milieu.* Cytokine Growth Factor Rev, 2001. **12**(1): p. 53-72.
37. Trinchieri, G., *Interleukin-12 and the regulation of innate resistance and adaptive immunity.* Nat Rev Immunol, 2003. **3**(2): p. 133-46.
38. Dupuis, S., et al., *Impaired response to interferon-alpha/beta and lethal viral disease in human STAT1 deficiency.* Nat Genet, 2003. **33**(3): p. 388-91.
39. Seth, R.B., L. Sun, and Z.J. Chen, *Antiviral innate immunity pathways.* Cell Res, 2006. **16**(2): p. 141-7.
40. Stockinger, B. and M. Veldhoen, *Differentiation and function of Th17 T cells.* Curr Opin Immunol, 2007. **19**(3): p. 281-6.
41. Watford, W.T., et al., *The biology of IL-12: coordinating innate and adaptive immune responses.* Cytokine Growth Factor Rev, 2003. **14**(5): p. 361-8.

42. Harrington, L.E., P.R. Mangan, and C.T. Weaver, *Expanding the effector CD4 T-cell repertoire: the Th17 lineage.* Curr Opin Immunol, 2006. **18**(3): p. 349-56.
43. Kamiya, S., et al., *An indispensable role for STAT1 in IL-27-induced T-bet expression but not proliferation of naive CD4+ T cells.* J Immunol, 2004. **173**(6): p. 3871-7.
44. Dong, C., *Diversification of T-helper-cell lineages: finding the family root of IL-17-producing cells.* Nat Rev Immunol, 2006. **6**(4): p. 329-33.
45. Hwang, E.S., et al., *T helper cell fate specified by kinase-mediated interaction of T-bet with GATA-3.* Science, 2005. **307**(5708): p. 430-3.
46. van der Geer, P., T. Hunter, and R.A. Lindberg, *Receptor protein-tyrosine kinases and their signal transduction pathways.* Annu Rev Cell Biol, 1994. **10**: p. 251-337.
47. Mescher, M.F., et al., *Molecular basis for checkpoints in the CD8 T cell response: tolerance versus activation.* Semin Immunol, 2007. **19**(3): p. 153-61.
48. Nguyen, V.A., et al., *Interferons activate the p42/44 mitogen-activated protein kinase and JAK-STAT (Janus kinase-signal transducer and activator transcription factor) signalling pathways in hepatocytes: differential regulation by acute ethanol via a protein kinase C-dependent mechanism.* Biochem J, 2000. **349**(Pt 2): p. 427-34.
49. Contursi, C., et al., *IFN consensus sequence binding protein potentiates STAT1-dependent activation of IFNgamma-responsive promoters in macrophages.* Proc Natl Acad Sci U S A, 2000. **97**(1): p. 91-6.
50. Ramana, C.V., et al., *Stat1-dependent and -independent pathways in IFN-gamma-dependent signaling.* Trends Immunol, 2002. **23**(2): p. 96-101.
51. Takaoka, A., et al., *Cross talk between interferon-gamma and -alpha/beta signaling components in caveolar membrane domains.* Science, 2000. **288**(5475): p. 2357-60.
52. Camoglio, L., et al., *Hapten-induced colitis associated with maintained Th1 and inflammatory responses in IFN-gamma receptor-deficient mice.* Eur J Immunol, 2000. **30**(5): p. 1486-95.
53. Haring, J.S. and J.T. Harty, *Aberrant contraction of antigen-specific CD4 T cells after infection in the absence of gamma interferon or its receptor.* Infect Immun, 2006. **74**(11): p. 6252-63.
54. Wurster, A.L., T. Tanaka, and M.J. Grusby, *The biology of Stat4 and Stat6.* Oncogene, 2000. **19**(21): p. 2577-84.
55. Rao, A. and O. Avni, *Molecular aspects of T-cell differentiation.* Br Med Bull, 2000. **56**(4): p. 969-84.
56. Borner, C., V. Hollt, and J. Kraus, *Cannabinoid receptor type 2 agonists induce transcription of the mu-opioid receptor gene in Jurkat T cells.* Mol Pharmacol, 2006. **69**(4): p. 1486-91.
57. Usui, T., et al., *T-bet regulates Th1 responses through essential effects on GATA-3 function rather than on IFNG gene acetylation and transcription.* J Exp Med, 2006. **203**(3): p. 755-66.
58. Young, H.A., *Unraveling the pros and cons of interferon-gamma gene regulation.* Immunity, 2006. **24**(5): p. 506-7.
59. McCune, K., et al., *Prognosis of hormone-dependent breast cancers: implications of the presence of dysfunctional transcriptional networks activated by insulin via the immune transcription factor T-bet.* Cancer Res, 2010. **70**(2): p. 685-96.
60. Dinarello, C.A., *Interleukin-18 and the pathogenesis of inflammatory diseases.* Semin Nephrol, 2007. **27**(1): p. 98-114.
61. Akira, S., *The role of IL-18 in innate immunity.* Curr Opin Immunol, 2000. **12**(1): p. 59-63.
62. Ozaki, K. and W.J. Leonard, *Cytokine and cytokine receptor pleiotropy and redundancy.* J Biol Chem, 2002. **277**(33): p. 29355-8.

63. Haque, S.J., P.C. Harbor, and B.R. Williams, *Identification of critical residues required for suppressor of cytokine signaling-specific regulation of interleukin-4 signaling.* J Biol Chem, 2000. **275**(34): p. 26500-6.
64. Loke, P. and J.P. Allison, *Emerging mechanisms of immune regulation: the extended B7 family and regulatory T cells.* Arthritis Res Ther, 2004. **6**(5): p. 208-14.
65. Guntermann, C. and D.R. Alexander, *CTLA-4 suppresses proximal TCR signaling in resting human CD4(+) T cells by inhibiting ZAP-70 Tyr(319) phosphorylation: a potential role for tyrosine phosphatases.* J Immunol, 2002. **168**(9): p. 4420-9.
66. Walton, K.M. and J.E. Dixon, *Protein tyrosine phosphatases.* Annu Rev Biochem, 1993. **62**: p. 101-20.
67. Pingel, S., et al., *The CD45 tyrosine phosphatase regulates CD3-induced signal transduction and T cell development in recombinase-deficient mice: restoration of pre-TCR function by active p56(lck).* Eur J Immunol, 1999. **29**(8): p. 2376-84.
68. Sato, T., et al., *Beta-selection of immature thymocytes is less dependent on CD45 tyrosinephosphatase.* Immunol Lett, 1998. **64**(2-3): p. 133-8.
69. Del Real, G., et al., *Blocking of HIV-1 infection by targeting CD4 to nonraft membrane domains.* J Exp Med, 2002. **196**(3): p. 293-301.
70. Woods, M.L., et al., *A novel function for the Tec family tyrosine kinase Itk in activation of beta 1 integrins by the T-cell receptor.* EMBO J, 2001. **20**(6): p. 1232-44.
71. Yablonski, D., et al., *A Nck-Pak1 signaling module is required for T-cell receptor-mediated activation of NFAT, but not of JNK.* EMBO J, 1998. **17**(19): p. 5647-57.
72. Gulbins, E., et al., *Direct stimulation of Vav guanine nucleotide exchange activity for Ras by phorbol esters and diglycerides.* Mol Cell Biol, 1994. **14**(7): p. 4749-58.
73. Han, J., et al., *Role of substrates and products of PI 3-kinase in regulating activation of Rac-related guanosine triphosphatases by Vav.* Science, 1998. **279**(5350): p. 558-60.
74. Woods, M.L. and Y. Shimizu, *Signaling networks regulating beta1 integrin-mediated adhesion of T lymphocytes to extracellular matrix.* J Leukoc Biol, 2001. **69**(6): p. 874-80.
75. Bogin, Y., et al., *SLP-76 mediates and maintains activation of the Tec family kinase ITK via the T cell antigen receptor-induced association between SLP-76 and ITK.* Proc Natl Acad Sci U S A, 2007. **104**(16): p. 6638-43.
76. Wiest, D.L., et al., *TCR activation of ZAP70 is impaired in CD4+CD8+ thymocytes as a consequence of intrathymic interactions that diminish available p56lck.* Immunity, 1996. **4**(5): p. 495-504.
77. Grader-Beck, T., et al., *cAMP inhibits both Ras and Rap1 activation in primary human T lymphocytes, but only Ras inhibition correlates with blockade of cell cycle progression.* Blood, 2003. **101**(3): p. 998-1006.
78. He, H.T. and D. Marguet, *T-cell antigen receptor triggering and lipid rafts: a matter of space and time scales. Talking Point on the involvement of lipid rafts in T-cell activation.* EMBO Rep, 2008. **9**(6): p. 525-30.
79. Lovatt, M., et al., *Lck regulates the threshold of activation in primary T cells, while both Lck and Fyn contribute to the magnitude of the extracellular signal-related kinase response.* Mol Cell Biol, 2006. **26**(22): p. 8655-65.
80. Palmieri, G., et al., *CD94/NKG2-A inhibitory complex blocks CD16-triggered Syk and extracellular regulated kinase activation, leading to cytotoxic function of human NK cells.* J Immunol, 1999. **162**(12): p. 7181-8.
81. Herndon, T.M., et al., *ZAP-70 and SLP-76 regulate protein kinase C-theta and NF-kappa B activation in response to engagement of CD3 and CD28.* J Immunol, 2001. **166**(9): p. 5654-64.

82. Frauwirth, K.A., et al., *The CD28 signaling pathway regulates glucose metabolism.* Immunity, 2002. **16**(6): p. 769-77.
83. Gibson, S., et al., *Efficient CD28 signalling leads to increases in the kinase activities of the TEC family tyrosine kinase EMT/ITK/TSK and the SRC family tyrosine kinase LCK.* Biochem J, 1998. **330 (Pt 3)**: p. 1123-8.
84. Qi, Q. and A. August, *Keeping the (kinase) party going: SLP-76 and ITK dance to the beat.* Sci STKE, 2007. **2007**(396): p. pe39.
85. Miller, A.T. and L.J. Berg, *New insights into the regulation and functions of Tec family tyrosine kinases in the immune system.* Curr Opin Immunol, 2002. **14**(3): p. 331-40.
86. Tsoukas, C.D., et al., *Itk/Emt: a link between T cell antigen receptor-mediated Ca2+ events and cytoskeletal reorganization.* Trends Immunol, 2001. **22**(1): p. 17-20.
87. Kornbluth, R.S. and G.W. Stone, *Immunostimulatory combinations: designing the next generation of vaccine adjuvants.* J Leukoc Biol, 2006. **80**(5): p. 1084-102.
88. Arch, R.H. and C.B. Thompson, *4-1BB and Ox40 are members of a tumor necrosis factor (TNF)-nerve growth factor receptor subfamily that bind TNF receptor-associated factors and activate nuclear factor kappaB.* Mol Cell Biol, 1998. **18**(1): p. 558-65.
89. Baumann, B., et al., *Constitutive IKK2 activation in acinar cells is sufficient to induce pancreatitis in vivo.* J Clin Invest, 2007. **117**(6): p. 1502-13.
90. Sasaki, Y., et al., *Canonical NF-kappaB activity, dispensable for B cell development, replaces BAFF-receptor signals and promotes B cell proliferation upon activation.* Immunity, 2006. **24**(6): p. 729-39.
91. Schroder, A.J., et al., *Cutting edge: STAT6 serves as a positive and negative regulator of gene expression in IL-4-stimulated B lymphocytes.* J Immunol, 2002. **168**(3): p. 996-1000.
92. Tailor, P., T. Tamura, and K. Ozato, *IRF family proteins and type I interferon induction in dendritic cells.* Cell Res, 2006. **16**(2): p. 134-40.
93. Nurieva, R.I., et al., *A costimulation-initiated signaling pathway regulates NFATc1 transcription in T lymphocytes.* J Immunol, 2007. **179**(2): p. 1096-103.
94. So, T., et al., *Signals from OX40 regulate nuclear factor of activated T cells c1 and T cell helper 2 lineage commitment.* Proc Natl Acad Sci U S A, 2006. **103**(10): p. 3740-5.
95. Jackson, J.G., Y.M. Usachev, and S.A. Thayer, *Bradykinin-induced nuclear factor of activated T-cells-dependent transcription in rat dorsal root ganglion neurons.* Mol Pharmacol, 2007. **72**(2): p. 303-10.
96. Skapenko, A., et al., *Antigen-independent Th2 cell differentiation by stimulation of CD28: regulation via IL-4 gene expression and mitogen-activated protein kinase activation.* J Immunol, 2001. **166**(7): p. 4283-92.
97. Appleman, L.J., et al., *CD28 costimulation mediates down-regulation of p27kip1 and cell cycle progression by activation of the PI3K/PKB signaling pathway in primary human T cells.* J Immunol, 2002. **168**(6): p. 2729-36.
98. Fos, C., et al., *ICOS ligation recruits the p50alpha PI3K regulatory subunit to the immunological synapse.* J Immunol, 2008. **181**(3): p. 1969-77.
99. Parry, R.V., et al., *CD28 and inducible costimulatory protein Src homology 2 binding domains show distinct regulation of phosphatidylinositol 3-kinase, Bcl-xL, and IL-2 expression in primary human CD4 T lymphocytes.* J Immunol, 2003. **171**(1): p. 166-74.
100. Kumar, R.N., et al., *Transactivation of platelet-derived growth factor receptor alpha by the GTPase-deficient activated mutant of Galpha12.* Mol Cell Biol, 2006. **26**(1): p. 50-62.

101. Lang, M.L., et al., *Fc alpha receptor cross-linking causes translocation of phosphatidylinositol-dependent protein kinase 1 and protein kinase B alpha to MHC class II peptide-loading-like compartments.* J Immunol, 2001. **166**(9): p. 5585-93.
102. Kane, L.P., et al., *Akt-dependent phosphorylation specifically regulates Cot induction of NF-kappa B-dependent transcription.* Mol Cell Biol, 2002. **22**(16): p. 5962-74.
103. Frost, J.A., et al., *Stimulation of NFkappa B activity by multiple signaling pathways requires PAK1.* J Biol Chem, 2000. **275**(26): p. 19693-9.
104. Cammarano, M.S. and A. Minden, *Dbl and the Rho GTPases activate NF kappa B by I kappa B kinase (IKK)-dependent and IKK-independent pathways.* J Biol Chem, 2001. **276**(28): p. 25876-82.
105. Swantek, J.L., L. Christerson, and M.H. Cobb, *Lipopolysaccharide-induced tumor necrosis factor-alpha promoter activity is inhibitor of nuclear factor-kappaB kinase-dependent.* J Biol Chem, 1999. **274**(17): p. 11667-71.
106. Van Gool, S.W., et al., *CD80, CD86 and CD40 provide accessory signals in a multiple-step T-cell activation model.* Immunol Rev, 1996. **153**: p. 47-83.
107. Ghoreschi, K., A. Laurence, and J.J. O'Shea, *Janus kinases in immune cell signaling.* Immunol Rev, 2009. **228**(1): p. 273-87.
108. Wu, C., et al., *SHP-1 suppresses cancer cell growth by promoting degradation of JAK kinases.* J Cell Biochem, 2003. **90**(5): p. 1026-37.
109. Losman, J.A., et al., *Cutting edge: SOCS-1 is a potent inhibitor of IL-4 signal transduction.* J Immunol, 1999. **162**(7): p. 3770-4.
110. Yasukawa, H., A. Sasaki, and A. Yoshimura, *Negative regulation of cytokine signaling pathways.* Annu Rev Immunol, 2000. **18**: p. 143-64.
111. Iwamoto, T., et al., *The JAK-inhibitor, JAB/SOCS-1 selectively inhibits cytokine-induced, but not v-Src induced JAK-STAT activation.* Oncogene, 2000. **19**(41): p. 4795-801.
112. Harada, N., et al., *Identification of the critical portions of the human IL-4 receptor alpha chain for activation of STAT6.* Biochem Biophys Res Commun, 1998. **246**(3): p. 675-80.
113. Pernis, A., et al., *Interleukin 4 signals through two related pathways.* Proc Natl Acad Sci U S A, 1995. **92**(17): p. 7971-5.
114. Heim, M.H., *The Jak-STAT pathway: cytokine signalling from the receptor to the nucleus.* J Recept Signal Transduct Res, 1999. **19**(1-4): p. 75-120.
115. Sirskyj, D., et al., *Disruption of the gamma c cytokine network in T cells during HIV infection.* Cytokine, 2008. **43**(1): p. 1-14.
116. Fenghao, X., et al., *Interleukin 4 activates a signal transducer and activator of transcription (Stat) protein which interacts with an interferon-gamma activation site-like sequence upstream of the I epsilon exon in a human B cell line. Evidence for the involvement of Janus kinase 3 and interleukin-4 Stat.* J Clin Invest, 1995. **96**(2): p. 907-14.
117. Korn, T., et al., *The dynamics of effector T cells and Foxp3+ regulatory T cells in the promotion and regulation of autoimmune encephalomyelitis.* J Neuroimmunol, 2007. **191**(1-2): p. 51-60.
118. Wahl, S.M., N. Vazquez, and W. Chen, *Regulatory T cells and transcription factors: gatekeepers in allergic inflammation.* Curr Opin Immunol, 2004. **16**(6): p. 768-74.
119. Verhagen, C.E., et al., *Residual type 1 immunity in patients genetically deficient for interleukin 12 receptor beta1 (IL-12Rbeta1): evidence for an IL-12Rbeta1-independent pathway of IL-12 responsiveness in human T cells.* J Exp Med, 2000. **192**(4): p. 517-28.

120. Lentsch, A.B., et al., *STAT4 and STAT6 regulate systemic inflammation and protect against lethal endotoxemia.* J Clin Invest, 2001. **108**(10): p. 1475-82.
121. Bour-Jordan, H., et al., *CTLA-4 regulates the requirement for cytokine-induced signals in T(H)2 lineage commitment.* Nat Immunol, 2003. **4**(2): p. 182-8.
122. Liu, Y., et al., *Unique expression of suppressor of cytokine signaling 3 is essential for classical macrophage activation in rodents in vitro and in vivo.* J Immunol, 2008. **180**(9): p. 6270-8.
123. de Hooge, A.S., et al., *Local activation of STAT-1 and STAT-3 in the inflamed synovium during zymosan-induced arthritis: exacerbation of joint inflammation in STAT-1 gene-knockout mice.* Arthritis Rheum, 2004. **50**(6): p. 2014-23.
124. Yu, C.R., et al., *Cell proliferation and STAT6 pathways are negatively regulated in T cells by STAT1 and suppressors of cytokine signaling.* J Immunol, 2004. **173**(2): p. 737-46.
125. Jackson, S.H., et al., *Dendritic cell maturation requires STAT1 and is under feedback regulation by suppressors of cytokine signaling.* J Immunol, 2004. **172**(4): p. 2307-15.
126. Daegelmann, C., et al., *Association between suppressors of cytokine signalling, T-helper type 1/T-helper type 2 balance and allergic sensitization in children.* Clin Exp Allergy, 2008. **38**(3): p. 438-48.
